# Proteomic Analysis of the Cyst Stage of *Entamoeba histolytica*

**DOI:** 10.1371/journal.pntd.0001643

**Published:** 2012-05-08

**Authors:** Ibne Karim M. Ali, Rashidul Haque, Abdullah Siddique, Mamun Kabir, Nicholas E. Sherman, Sean A. Gray, Gerard A. Cangelosi, William A. Petri

**Affiliations:** 1 Division of Infectious Disease and International Health, University of Virginia Health System, Charlottesville, Virginia, United States of America; 2 International Center for Diarrhoeal Disease Research Bangladesh, Mohakhali, Dhaka, Bangladesh; 3 W. M. Keck Biomedical Mass Spectrometry Core, University of Virginia Health Sciences Center, Charlottesville, Virginia, United States of America; 4 Seattle Biomedical Research Institute, Seattle, Washington, United States of America; Jawaharlal Nehru University, India

## Abstract

**Background:**

The category B agent of bioterrorism, *Entamoeba histolytica* has a two-stage life cycle: an infective cyst stage, and an invasive trophozoite stage. Due to our inability to effectively induce encystation *in vitro*, our knowledge about the cyst form remains limited. This also hampers our ability to develop cyst-specific diagnostic tools.

**Aims:**

Three main aims were (i) to identify *E. histolytica* proteins in cyst samples, (ii) to enrich our knowledge about the cyst stage, and (iii) to identify candidate proteins to develop cyst-specific diagnostic tools.

**Methods:**

Cysts were purified from the stool of infected individuals using Percoll (gradient) purification. A highly sensitive LC-MS/MS mass spectrometer (Orbitrap) was used to identify cyst proteins.

**Results:**

A total of 417 non-redundant *E. histolytica* proteins were identified including 195 proteins that were never detected in trophozoite-derived proteomes or expressed sequence tag (EST) datasets, consistent with cyst specificity. Cyst-wall specific glycoproteins Jacob, Jessie and chitinase were positively identified. Antibodies produced against Jacob identified cysts in fecal specimens and have potential utility as a diagnostic reagent. Several protein kinases, small GTPase signaling molecules, DNA repair proteins, epigenetic regulators, and surface associated proteins were also identified. Proteins we identified are likely to be among the most abundant in excreted cysts, and therefore show promise as diagnostic targets.

**Major Conclusions:**

The proteome data generated here are a first for naturally-occurring *E. histolytica* cysts, and they provide important insights into the infectious cyst form. Additionally, numerous unique candidate proteins were identified which will aid the development of new diagnostic tools for identification of *E. histolytica* cysts.

## Author Summary

We used tandem mass spectrometry to identify *E. histolytica* cyst proteins in 5 cyst positive stool samples. We report the identification of 417 non-redundant *E. histolytica* proteins including 195 proteins that were not identified in existing trophozoite derived proteome or EST datasets, consistent with cyst specificity. Because the cysts were derived directly from patient samples with incomplete purification, a limited number of proteins were identified (N = 417) that probably represent only a partial proteome. Nevertheless, the study succeeded in identifying proteins that are likely to be abundant in the cyst stage of the parasite. Several of these proteins may play roles in *E. histolytica* stage conversion or cyst function. Proteins identified in this study may be useful markers for diagnostic detection of *E. histolytica* cysts. Overall, the data generated in this study promises to aid the understanding of the cyst stage of the parasite which is vital for disease transmission and pathogenesis in *E. histolytica*.

## Introduction

The parasitic protozoan *Entamoeba histolytica* is the causative agent of amebic colitis and amebic liver abscesses in humans [1], [2]. The World Health Organization estimates up to 50 million invasive infections world-wide annually [3]. *E. histolytica* has a simple, two-stage life cycle, consisting of the infective cyst and colon-invasive trophozoite forms. *E. histolytica* infections occur when cysts are ingested through contaminated food or water. In the lower intestine trophozoites emerge from cysts (a process known as excystation). As a result of unknown stimuli in the intestine, trophozoites again can differentiate into cysts (a process known as encystation), which may be excreted in feces to infect other humans. Although the cyst is the only form to transmit infections, most studies on *E. histolytica* have focused on the trophozoite form, which is the only form that can be readily cultured. The inability to encyst trophozoites *in vitro* has severely impaired our knowledge on the infectious stage of *E. histolytica*.

There is an increasing recognition of the burden of infection due to this protozoan parasite. Carefully conducted serologic studies in Mexico, where amebiasis is endemic, demonstrated antibody to *E. histolytica* in 8.4% of the population [4]. In the urban slum of Fortaleza, Brazil, 25% of the people tested carried antibody to *E. histolytica*; the prevalence of anti-amebic antibodies in children aged six to fourteen years was 40% [5]. A prospective study of preschool children in a slum of Dhaka, Bangladesh demonstrated new *E. histolytica* infection in 39% of children over a one year period of observation, with 10% of the children having an *E. histolytica* infection associated with diarrhea and 3% with dysentery [6].

The diagnosis of *E. histolytica* infection in endemic areas still relies on microscopy, which is neither sensitive nor specific [7]. PCR-based diagnostic methods have not replaced microscopy in endemic areas, as they require skilled people and sophisticated laboratory settings which are absent in these areas. Although there are simple (ELISA-based) diagnostic tools available to detect the trophozoite form of *E. histolytica*, none are designed to detect the cyst form of parasite. There are two reasons to produce tests that detect the cyst form of *E. histolytica*. First, the cyst is the infectious form of the parasite, and therefore of greatest importance for detection in potential food- or water-borne outbreaks, both natural and man-made. Second, cyst antigens are expected to be more abundant, stable, and readily detectable in stool samples, including formalin-preserved samples. Lack of such stability is the major limitation of the current *E. histolytica* antigen-detection test by TechLab [8]. However our understanding of *E. histolytica* cyst proteins remains the major factor limiting our ability to develop cyst specific diagnostic reagents.

Relatively more is known about the cyst stage of the reptilian parasite *Entamoeba invadens* [9]. *E. invadens* can be induced to encyst *in vitro* [10]–[12], a phenomenon that has made it a model system to study encystation. Studies have identified osmotic shock [13], low glucose [12] or carbon source [14], and levels of serum and mucin [15] as potential triggers of encystation in *E. invadens in vitro*. Additionally, cyst- and cyst-wall specific genes have been identified including chitin synthetase [16], chitinase [17], chitosan [18], and the lectins Jessie and Jacobs [19]. A 70-kDa heat shock protein [20], and proteins involved in proteasome function [21] were also found to be linked to encystation in *E. invadens*.

*E. histolytica* strains can undergo spontaneous encystation, although very inefficiently, when grown in presence of bacteria [22]. A pioneering microarray analysis of this process identified about 15% of all genes in the genome as developmentally regulated based on their mRNA transcript levels (>3-fold change, p-value<0.01) including 672 genes referred to as cyst-specific and 767 genes referred to as trophozoite-specific. The cyst-specific genes included cysteine proteases, putative DNA-binding or transcription factor-related proteins (such as Myb domain proteins) and signal transduction-related transmembrane protein kinases. The promoter motif for one of the Myb domain proteins was later characterized for and this motif appeared to function in the regulation of a subset of cyst-specific genes [23].

In contrast to transcriptomic data, no proteomic data are currently available for *E. histolytica* cysts. Proteomic analysis of cysts and trophozoites of *E. invadens* using high resolution 2D PAGE and digitized video image analysis of silver stained gels identified a total of 155 proteins unique to trophozoites and a total of 72 proteins unique to cysts [24]. Lack of knowledge of the *E. histolytica* cyst proteome hampers our ability to develop cyst-targeted diagnostic tools and to investigate stage conversion in this parasite. In this study, we applied mass spectrometry based whole genome shotgun sequencing approaches to 5 purified cyst samples in order to identify proteins expressed in *E. histolytica* cysts *in vivo*. Here we present for the first time the identification of 417 proteins (representing ∼5.1% of all 8201 predicted proteins) in the purified cyst samples. Out of the 417 proteins identified, 195 have never been detected in the *E. histolytica* trophozoite specific proteome or EST databases. This is the first proteomic analysis of naturally-occurring *E. histolytica* cysts.

## Methods

### Ethics statement

The collection of clinical samples used in this study has been approved by the Ethical Review Committee (ERC) of ICDDR,B. Since the study participants were minors (aged 2–6 years), parents or legal guardians provided written informed consent on behalf of all child participants. All clinical investigation has been conducted according to the principles expressed in the Declaration of Helsinki.

### Screening of *E. histolytica* cysts

Fecal specimens were collected from children enrolled in an ongoing field study of human immunity to amebiasis in an urban slum community at Mirpur, Dhaka, Bangladesh. They were screened for the presence of *E. histolytica* cysts in their stools. Initially, fresh stool samples were checked by microscopy for cysts of *E. histolytica/E. dispar/E. moshkovskii*. Positive stools were then subject to *E. histolytica*-specific antigen detection by *E. histolytica*-II ELISA (TechLab, Blackburg, VA) as described previously [25]. Since this ELISA was incapable of distinguishing between a mixed infection of *E. histolytica/E. dispar* and a single infection of *E. histolytica*, ELISA positive stool samples were further subject to DNA purification and diagnostic PCR for *E. histolytica* and *E. dispar*. PCR was also performed for *E. moshkovskii*-specific DNA. Cysts were only purified from stool samples that were PCR positive solely for *E. histolytica*.

### Cyst purification

Cysts were purified from stool samples as described previously with some modifications [26]–[28]. In brief, about 4–5 g of stool was dissolved in PBS, filtered through two layers of gauges, centrifuged at 3220×g for 20 minutes, and the supernatant discarded. After washing 3 times with PBS at 3220×g for 20 minutes, the pellet was suspended in 5 mL of ethyl acetate (to separate the cyst and the fecal debris) and centrifuged at 3220×g for 20 minutes. The supernatant was discarded and the pellet was washed 3 more times with PBS (at 3220×g for 20 minutes), and the pellet was resuspended in 2 mL of PBS. This was then carefully layered using a Pasteur pipette on top of a previously prepared 10–80% Percoll gradient in 17×120 mm, 15 mL high-clarity polypropylene conical tubes by layering 2 mLs each of 80% Percoll (in PBS), followed by 50%, 40%, 30%, 20% and 10% Percoll solutions. This was centrifuged at 3220×g for 20 minutes. The content between 80% and 40% of the Percoll gradient was transferred to a new tube, washed 3 times with PBS and checked by microscopy for purified cysts.

### Production of polyclonal antibodies against Jacob

The rabbit sera developed against one member of *E. histolytica* Jacob (EHI_044500), which shows 83.1% identity and 88.1% similarity with the *E. dispar* Jacob at amino acid level, were purchased from the Cocalico Biologicals, Inc. (project number 2010-0158). This protocol has been approved by the Animal Care and Use Committee of Cocalico Biologicals, Inc. who follows the USDA and NIH guidelines. The Office of the Laboratory of Animal Welfare, Division of Assurance, National Institute of Health has approved the Animal Welfare Assurance (#A3669-01) of Cocalico Biologicals, Inc.

### Fluorescence microscopy with the purified cysts

About 50 µl of the *E. histolytica* cysts purified using the Percoll gradient centrifugation were fixed on a microscopic slide by adding 4% formaldehyde solution for 1 hour at 37°C. After washing with 1× PBS containing 0.1% Triton X-100, cysts were treated with either 1∶200 diluted post-immune or pre-immune antisera against *E. histolytica* cyst-wall specific Jacob protein raised in rabbits. They were then stained with 1∶200 diluted Fluorescein isothiocyanate (FITC)-conjugated goat anti-rabbit secondary antibody and examined by immunofluorescence microscopy.

### Preparation of *E. histolytica* proteins from the purified cysts

Crude proteins from purified cyst samples were isolated in either of two ways. In the first protocol, cysts were subject to 5 freeze-and-thaw cycles to facilitate the breakage of cyst wall, followed by 20–25 minutes of sonication (30-second pulse followed by 30-second rest) in presence of protease inhibitor (Sigma-Aldrich). This procedure was followed for only one sample, AM951. In the second protocol, cysts were subject to 20–25 minutes of sonication (30-second pulse followed by 30-second rest) in presence of protease inhibitor (Sigma-Aldrich) eliminating the first freeze-and-thaw cycles as above. This procedure was followed for all 5 samples, including AM951. Protein concentration was measured using the Bio-Rad Protein Assay (Bio-Rad, USA) according to the manufacturer's instructions.

### Mass spectrometry

All the cyst samples (after extraction of crude proteins) were divided into two parts (except for one, sample 4268, which had too little protein to divide into two parts) – supernatant (which should contain soluble proteins) and pellet (which should contain insoluble proteins).

The sample supernatant was removed, dried, and suspended in SDS-PAGE loading buffer. The remaining pellet was soaked in SDS-PAGE loading buffer. For sample 4268, the whole sample (containing both the supernatant and the beads) was dried, and suspended in SDS-PAGE loading buffer. They were loaded on a gel and run until the dye front was ∼1 cm into the gel. This 1 cm×1 cm area of gel was cut for processing (2 gel pieces for each sample, except sample 4268). For sample 4268, the entire sample (containing both the supernatant and the beads) was dried, suspended in SDS-PAGE loading buffer, and processed as above as single gel piece.

The gel piece for each sample was cubed and transferred to a siliconized tube and washed and destained in 200 µL 50% methanol for 4 h. The gel pieces were dehydrated in acetonitrile, rehydrated in 30 µL of 10 mM dithiolthreitol in 0.1 M ammonium bicarbonate and reduced at room temperature for 0.5 h. The DTT solution was removed and the sample alkylated in 30 µL 50 mM iodoacetamide in 0.1 M ammonium bicarbonate at room temperature for 0.5 h. The reagent was removed and the gel pieces dehydrated in 100 µL acetonitrile. The acetonitrile was removed and the gel pieces rehydrated in 100 µL 0.1 M ammonium bicarbonate. The pieces were dehydrated in 100 µL acetonitrile, the acetonitrile removed and the pieces completely dried by vacuum centrifugation. The gel pieces were rehydrated in 20 ng/µL trypsin in 50 mM ammonium bicarbonate on ice for 10 min. Any excess enzyme solution was removed and 20 µL 50 mM ammonium bicarbonate added. The sample was digested overnight at 37°C and the peptides formed extracted from the polyacrylamide in two 50 µL aliquots of 50% acetonitrile/5% formic acid. These extracts were combined and evaporated to 15 µL for MS analysis.

The LC-MS/MS system consisted of a Thermo Electron Orbitrap Velos ETD mass spectrometer system with a Protana nanospray ion source interfaced to a self-packed 8 cm×75 um id Phenomenex Jupiter 10 um C18 reversed-phase capillary column. Seven microliters of the extract was injected and the peptides eluted from the column by an acetonitrile/0.1 M acetic acid gradient at a flow rate of 0.5 µL/min over 1 hr. The nanospray ion source was operated at 2.5 kV. The digest was analyzed using the double play capability of the instrument acquiring a full scan mass spectrum (MS – Orbitrap 60K resolution) to determine peptide molecular weights and 20 product ion spectra (MS/MS – Ion trap) to determine amino acid sequence over the gradient elution.

### Mass spectrometry data analysis

The mass spectrometry data were analyzed by database searching using the Sequest search algorithm (Bioworks 3.3.1) against *E. histolytica* (downloaded from NCBI Oct 2010), IPI Human (Ver 3.78), and NCBI NR (downloaded Jan 2011). The data were loaded in Scaffold v3 and minimal filters were set (Peptide score >60%, Protein score >90%, Xcorr >1.8 (+1), 2.2 (+2), 2.5(+3) and 3.5 (+4 or greater)). The p-values were determined using the two-tailed Fisher's exact test.

## Results

### Five *E. histolytica* cyst positive samples were selected for proteomic study

In order to identify *E. histolytica* cyst positive samples, we performed microscopical examination of stool specimens from children enrolled in an ongoing study on amebiasis. However, since the cyst of *E. histolytica* is morphologically indistinguishable from that of *E. dispar* and *E. moshkovskii*, microscopy-positive samples were then subject to species-specific *E. histolytica* II ELISA (TechLab, Blacksburg, VA). One limitation of *E. histolytica* II ELISA is that it cannot differentiate between a co-infection of *E. histolytica* with *E. dispar* or *E. moshkovskii* and a single infection of *E. histolytica*. Therefore, microscopy/ELISA positive samples were then subject to *E. histolytica*-, *E. dispar*- and *E. moshkovskii*-specific PCRs. Using these methods, we identified 5 stool specimens that were positive only for *E. histolytica* (data not shown). Microscopy revealed that 4 of these samples had only the cyst form of the parasite, while the 5^th^ sample (4268) had both cyst and trophozoite forms in the original stool sample, although cysts were the predominant form. Five samples were derived from children aged 2–6 years, three of them were from males (4268, AM951, and AM797), and two of them were from females (8076 and CMS33-7132).

### Verification of cyst structures after Percoll purification

**Figure 1:**
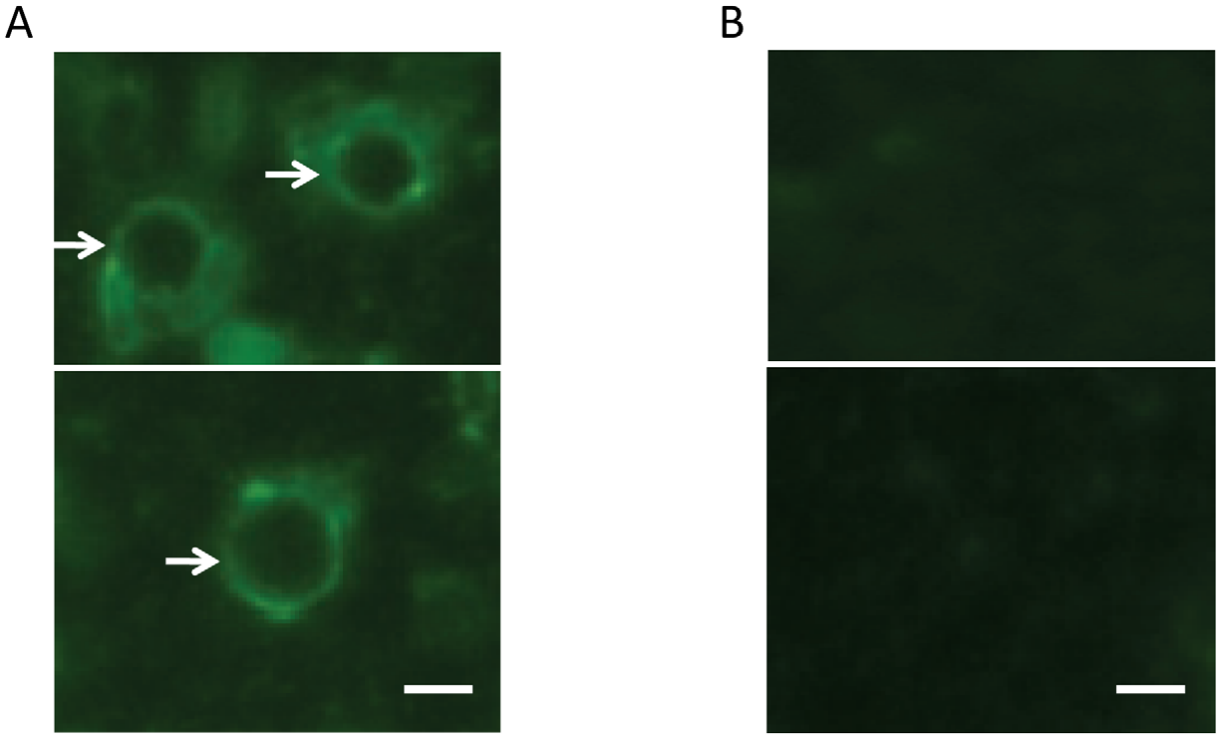
Detection of *Entamoeba histolytica* cysts in Percoll purified cyst samples by immunofluorescence to the Jacob protein. *E. histolytica* cysts were purified using the Percoll density gradient centrifugation [26]–[28], and about 50 µL of the purified cysts were fixed in microscopic slides by adding 4% formaldehyde solution for 1 hour at 37°C. After washing with 1× PBS containing 0.1% Triton X-100, cells were treated with either 1∶200 diluted post-immune or pre-immune antisera against *E. histolytica* cyst-wall specific Jacob protein raised in rabbits. They were then labeled with 1∶200 diluted FITC-conjugated goat anti-rabbit secondary antibody and examined by immunofluorescence microscopy. (a) Anti-Jacob antisera stained the cyst wall (shown by white arrows), (b) while pre-immune antisera failed to stain cyst wall. White bar is approximately 10 microns.

Following a positive microscopical identification, Percoll purification was used to significantly enrich the cyst form of the parasite (data not shown). In order to verify that the cyst-like structures in the cyst sample after Percoll purification were indeed *E. histolytica* cysts, we performed immunofluorescence assay using a polyclonal antibody developed in rabbits against a highly abundant cyst protein Jacob (EHI_044500). While the rabbit preimmune sera could not recognize cyst structures, the immune sera clearly stained the round-shaped cyst wall of *E. histolytica* in the Percoll-purified sample as expected (Figure 1).

In order to investigate whether the Percoll gradient cyst purification protocol may have also co-purified the trophozoite form of the parasite, we added 0, 100, and 1000 *E. histolytica* trophozoites into 1 g each of three *E. histolytica*-negative stool samples (by microscopy, culture, ELISA and PCR), and they were subject to identical Percoll gradient cyst purification as performed with the five test samples. We then performed *E. histolytica* II ELISA using the contents from the 40–80% fractions of Percoll gradient to detect lectin antigen (which expresses most abundantly in the trophozoite form), and found that all three samples were negative for *E. histolytica* lectin (data not shown). This suggested that the Percoll gradient purification of stool specimens selectively enriched cysts but not trophozoites. Since only 1 out of 5 cyst samples (ID: 4268) had microscopically positive trophozoite in the initial stool examination, and the number of trophozoites in this sample was much smaller than that of cysts (data not shown), we conclude that our proteomic data was comprised predominantly (if not entirely) of proteins expressed in cysts.

In order to determine which method of lysate preparation worked best for mass spectrometry analysis, we applied two methods on 1 of the 5 cyst samples (ID: AM951). The first method was sonication only, while the second method employed freeze-thaw followed by sonication. Mass spectrometry results suggested that a simple sonication-only approach was able to identify more proteins than an alternative approach involving a prior freeze-thaw step. Samples processed by sonication yielded about 20% more identifiable *E. histolytica* proteins (total 101) than samples processed by the freeze-thaw-sonication approach, which yielded only 81 identifiable *E. histolytica* proteins. Fifty-two proteins overlapped between the two approaches (Tables S1 and S2). We investigated if there were any qualitative differences in proteins identified by the two different methods of lysate preparation. Two calcium binding or signal related proteins, and two cyst wall specific proteins were detected in both methods of lysate preparations (Table S2). In contrary, out of 3 DNA repair proteins 2 were identified in the sonication only method and the third one was identified by freeze-thaw-sonication method only. The only surface associated protein (EHI_065330) was isolated by the sonication only method. Proportionately more proteins with putative enzymatic function were identified by freeze-thaw-sonication (19/81 or 23.5%) compared to sonication only (18/101 or 17.8%). In contrast, slightly more actin-related proteins were identified in sonication only method (7/101 or 6.9%) compared to freeze-thaw-sonication method (3/81 or 3.7%). However, these differences were not statistically significant. So, we conclude that there was no particular trend that could be detected in differentiating protein categories identified by sonication only or freeze-thaw-sonication methods.

Mass spectrometry was carried out for 4 out of 5 cyst samples in such a way as to detect both soluble and insoluble proteins (for details, see the materials and methods section). For the 5^th^ sample (4268), there was not enough cyst material to separate soluble and insoluble proteins and the two fractions were analyzed together. In most cases more proteins were identified in insoluble fraction than soluble fraction (59/48 for AM951, 99/42 for AM797, and 57/35 for CMS33-7132; Figure S1).

### Identification of *E. histolytica* proteins in cyst samples

**Figure 2:**
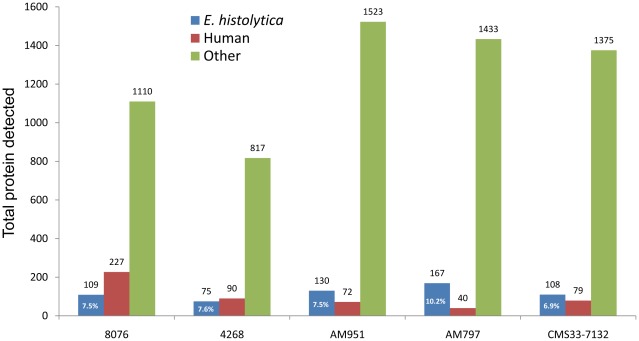
Total number of *E. histolytica*, human and other proteins detected in 5 cyst samples. In 5 cyst samples, the total number of proteins detected by MS/MS analysis ranged from 982 (in sample 4268) to 1725 (in sample AM951). The maximum number of *E. histolytica* proteins detected in a sample was 167 (sample AM797), while the least number of *E. histolytica* protein was 75 (sample 4268). The percentages of total proteins belong to *E. histolytica* are shown inside the bar graphs for each sample, which ranged from 6.9% (for sample CMS33-7133 to 10.2% (for sample AM797).

A total of 417 unique *E. histolytica* proteins were identified in the 5 cyst preparations. Human, bacterial, and rice-related proteins comprised about 90% of all proteins identified in the purified cyst samples (Figure 2). Human and rice-related proteins were abundant because of the source of cyst material (human feces) and nutritional habit of people in that region of the world (Dhaka, Bangladesh). Bacteria attached to the surfaces of cysts or inside the cysts might be the predominant source of bacterial proteins in the proteomic data. The proportions of human or rice related proteins may be reduced by using more stringent washing steps (from large amount of initial stool volume). However, cyst protein yields would be reduced and it would be very difficult, if not impossible, to get rid of bacteria that are attached to or ingested by the cysts. The 5 cyst samples came from 5 children aged 2–6 years, as a result, the amount of original stools that could be collected was very low, which was a limitation of this work. Nevertheless, *E. histolytica* proteins were identified in each of the 5 cyst samples as expected. The percentage of total proteins identified that were *E. histolytica* proteins in various cyst samples ranged from 6.9 to 10.2% (Figure 2).

### Comparison of proteomic data with other *E. histolytica* data

**Figure 3:**
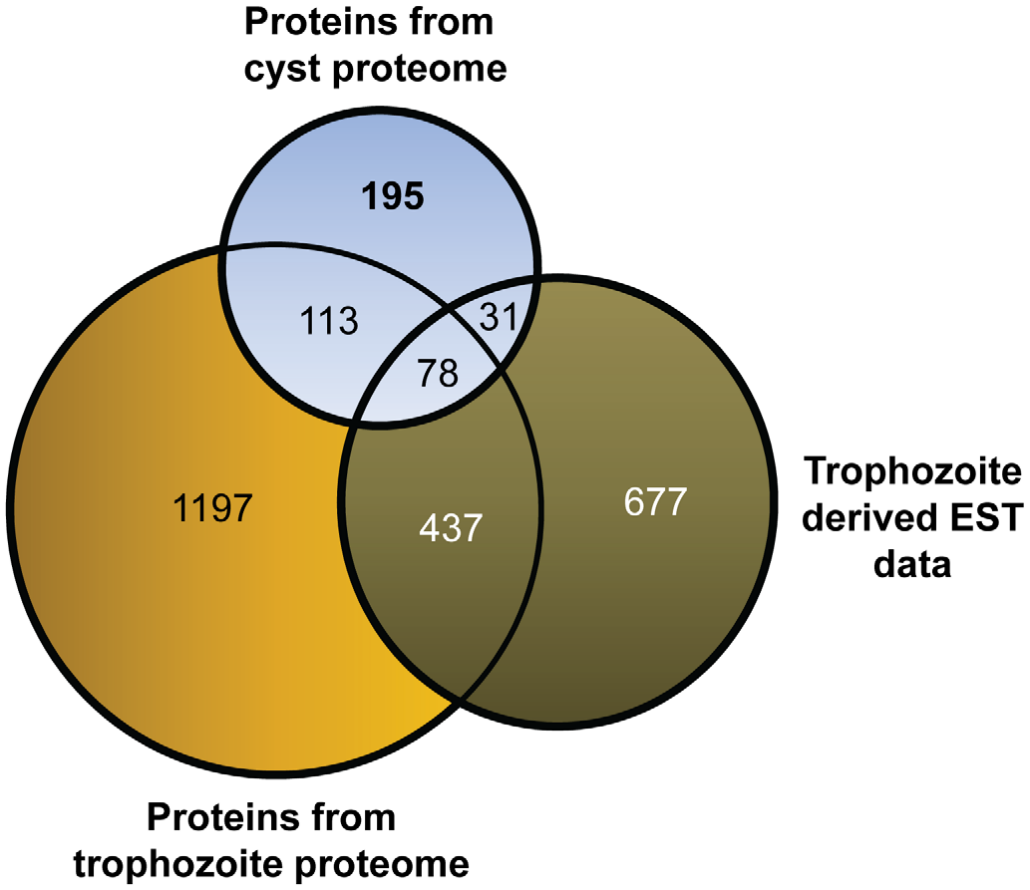
Venn diagram for 3-way comparison between the proteins of this study, proteins from the trophozoite proteome and the EST data on trophozoite. Overlaps between the *E. histolytica* cyst proteomic data (N = 417), trophozoite proteomic data (N = 1825), and the trophozoite specific EST data (N = 1223) were checked. A total of 191 proteins overlapped between the cyst proteomic and trophozoite proteomic datasets. A total of 109 proteins overlapped between the cyst proteomic and the trophozoite EST datasets. Seventy eight proteins overlapped between all three datasets. A total of 195 proteins of cyst proteomic study did not show any overlaps with other two datasets derived from trophozoites of *E. histolytica*, suggesting these are *E. histolytica* cyst-specific proteins.

Since the total number of proteins identified in the naturally-occurring cyst samples was small, we asked how unique these proteins are in comparison with the publicly available protein datasets in AmoebaDB (http://amoebadb.org/amoeba/). Two LC-MS/MS proteomic datasets were available for the trophozoite stage of *E. histolytica* comprising a total of 1825 proteins (as of November 30, 2011) [29], [30]. There was also available an EST database for *E. histolytica* trophozoite comprising a total of 1223 proteins (as of October 1, 2011; from a total of 20,812 public entries at http://www.ncbi.nlm.nih.gov/dbEST/dbEST_summary.html). A three-way comparison of protein overlaps between the three datasets (i.e. cyst proteome, trophozoite proteome, and trophozoite EST) was done using the freely available software in the internet (http://www.cmbi.ru.nl/cdd/biovenn/index.php) [31]. The Venn diagram shows that a total of 191 proteins overlapped between the cyst proteomic and trophozoite proteomic (LC-MS/MS) datasets, 109 proteins overlapped between the cyst proteomic and trophozoite EST datasets, and 78 proteins overlapped between all three datasets (Figure 3). It was surprising to see that only 515 proteins overlapped between the trophozoite proteome (515/1825 or 28.2%) and the EST datasets (515/1223 or 42.1%). This disparity is probably because only limited numbers of trophozoite-derived proteome data are available for *E. histolytica*. The two proteome works available at the AmoebaDB public database when this manuscript was being written (November 2011) were highly specific in nature - one attempted to identify trophozoite proteins from phagosome [29], while the other attempted to identify Concavalin A-enriched glycoproteins from the *E. histolytica* trophozoite [30]. So, we think many of the trophozoite-specific proteins are not represented in them, resulted in showing poor overlap with the trophozoite-derive EST datasets from *E. histolytica*. Nevertheless, a unique set of 195 proteins in the cyst proteomic dataset did not overlap with the other two datasets. This set might include *E. histolytica* cyst-specific proteins.

The cyst proteomic data was also compared with the only available mRNA transcriptome data for encysting cultures grown *in vitro* [22]. The encysting transcriptome was performed on recently isolated *E. histolytica* strains that can spontaneously encyst *in vitro* when grown in complex diphasic Robinson's medium. Six hundred and seventy two genes were identified to be up-regulated in *E. histolytica* cysts (based on ≥3-fold expression change and p-value of <0.05). Out of 417 cyst proteins detected in this study, 23 had no mRNA expression data available. Forty-seven of the remaining 394 proteins identified here overlapped with the 672 cyst-specific genes identified by transcriptomics (p-value 0.0058) (Figure S2). We then asked whether the unique set of 195 proteins that did not overlap with the trophozoite proteomic and the EST datasets exhibited a stronger overlap with the 672 cyst-specific genes. Out of 195 unique cyst proteins, 10 had no mRNA expression data available. However, 28 of the remaining 185 proteins identified by cyst proteomic analysis overlapped with the 672 cyst-specific genes identified by transcriptomic analysis. As expected, this overlap between cyst-specific transcripts and unique cyst proteins was stronger (p-value 0.0014) than the overlap between cyst-specific transcripts and cyst proteins in general (p-value 0.0058) (Figure S3). Despite the fact that there was a statistically significant overlap between the cyst-specific transcripts [22] and cyst proteins of this study, we did notice a large discrepancy between the two datasets. Several factors could explain this discrepancy: (i) our cyst proteomic data was based on naturally occurring cyst samples, while the Ehrenkaufer and colleague's cyst transcriptome data was based on cyst-like cultures obtained *in vitro*; (ii) the number of proteins identified in the cyst proteome was very low, and they represent only ∼5.1% (417/8201) of all proteins; (iii) cyst-specific genes were categorized based on >3-fold higher mRNA levels compared to that of trophozoites, as a result, there will be some proteins that are expressed in lower (>3-fold or more) levels in cysts compared to trophozoites, but will still be regarded as cyst-specific by Ehrenkaufer et al; and (iv) some mRNA transcript levels may not correspond to protein levels due to post-transcriptional regulation or protein degradation.

### Comparison of proteomic data with the *Entamoeba invadens* data

The cyst proteomic data was also compared with available data from developmental studies on the reptilian species *E. invadens*, whose encystation can be induced easily *in vitro*. Two *E. histolytica* chitinases, EHI_109890 and EHI_152170, show 79% and 47% identities with the *E. invadens* chitinases, EiChit1 and EiChit4, respectively [32]. In *E. invadens*, the mRNA expression of 4 chitinases was studied by Makioka and colleagues [32] during *in vitro* encystation and excystation. In the early phase of encystation, mRNA expression of all 4 chitinases increased although the greatest increase was seen for EiChit1 and EiChit4. However, following 5 hours of excystation, mRNA levels of these two chitinases dropped sharply compared to pre-induction stage, suggesting that these are highly cyst-specific. Both of these chitinase homologues (EHI_109890 and EHI_152170) were detected in our cyst samples as expected. Three actin depolymerizing factor (ADF) family proteins have been identified in *E. invadens* [33]. These proteins are thought to be important in actin cytoskeleton reorganization during development of *E. invadens*, and can be detected in both trophozoite and cyst stages of this parasite. The single member of ADF family protein in *E. histolytica* (actophorin/EHI_197480), which shows virtually no mRNA differences between trophozoite and cyst stages (Table S3; [22]) was identified in our cyst proteome, consistent with a similar role in the development of *E. histolytica*. An *E. histolytica* glycolytic enzyme enolase (EHI_130700) shows 85% identity with the *E. invadens* enolase (EIN_093390). This protein was present in the cytoplasmic vesicles as well as in the cyst wall of *E. invadens* as revealed by immunofluorescence microscopy [34]. However, this protein was present only in the cytoplasmic vesicles of *E. histolytica* trophozoites recovered from amebic liver lesions of experimental animals [34]. Additionally, enolase was detected in the mature cyst wall of *E. histolytica* in samples derived from human infections. Consistent with this finding, we could also detect enolase in 3 out of 5 cyst samples in our proteomic study. An earlier study has shown that heat shock treatment of *E. invadens* can result in strong induction of cyst-specific chitinase and Jacob mRNAs, and moderate induction of heat shock protein mRNAs, including a 70-kDa heat shock protein known as BiP (AF252299) [20]. Although heat shock alone cannot produce matured, chitin-walled cysts, these authors suggest that amebic heat shock proteins are involved in degradation of cytoskeletal proteins during encystation. In our cyst proteome study, 5 putative heat shock proteins were detected (Table S3), and one of these (EHI_199590) is a homoologue of *E. invadens* BiP (with 88% identity), suggesting a similar role of heat shock proteins in *E. histolytica* encystation.

However, other proteins that appeared to be important in *E. invadens* development were not detected in our study. For example, the *E. histolytica* homologue of one of the cysteine proteases EiCP-B9 [35], or profilins (such as EiPFN1 and EiPFN4; [36]) that are expressed in the cyst stage of *E. invadens*, were not detected in our proteome study. These discrepancies could be due to the fact that we were only able to identify a fraction of all cyst-specific proteins. Alternatively, they might reflect differences between parasite species and/or between cysts generated in hosts and *in vitro*.

### Functional categories of proteins identified in the *E. histolytica* cyst preparations

**Figure 4:**
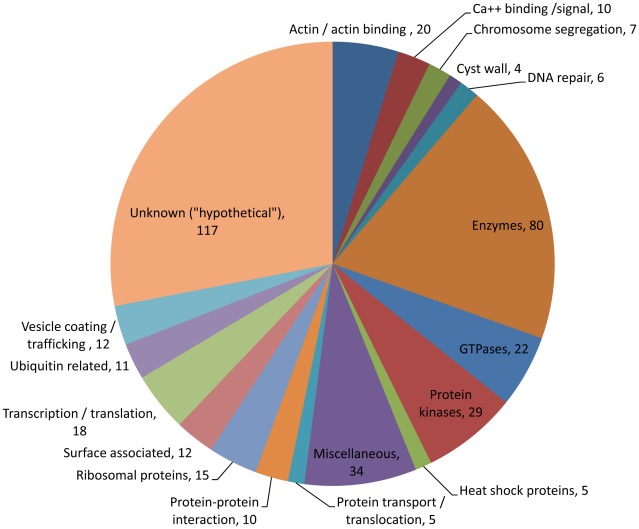
Categories of all 417 proteins detected in 5 cyst samples. *E. histolytica* proteins that were present in 1 or more cyst preparations fell into functional categories based on their annotations in the database. Out of 417 proteins, 117 did not have known function and are designated as hypothetical proteins. Four *E. histolytica* cyst-wall specific proteins (such as Jacob, two chitinases and chitin synthetase) were present in 1 or more cyst preparations. A total of 133 various enzymes (mostly protein kinases and GTPases) were also detected. Among other proteins, 18 transcription or translation related proteins (important for understanding of encystation biology), 12 surface associated proteins, 15 ribosomal proteins, 10 protein-protein interacting proteins, 12 vesicle coating or trafficking protein, 10 calcium signal related proteins, and 6 DNA repair proteins are notable.

**Figure 5:**
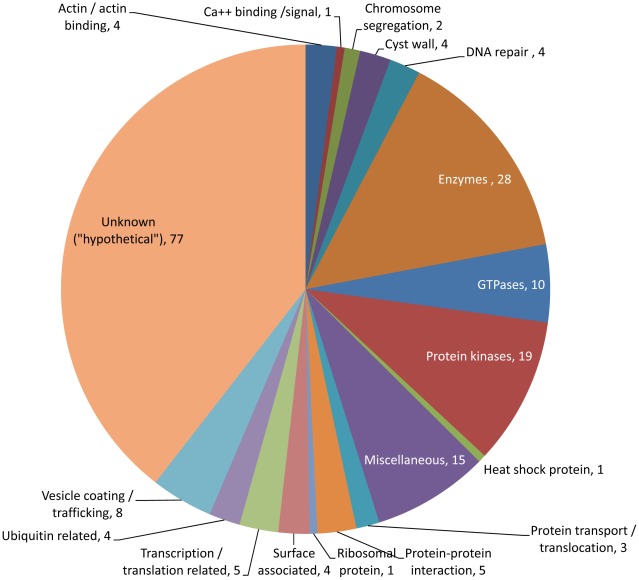
Categories of 195 unique cyst specific proteins. *E. histolytica* proteins that were present in 1 or more cyst preparations and those that were not found in trophozoite proteome or trophozoite EST datasets were categorized in various functional categories based on their annotations in the database. Out of a total 195 proteins, 77 did not have any known function, and they are designated as hypothetical proteins. Four *E. histolytica* cyst-wall specific proteins (such as Jacob, 2 chitinases and chitin synthetase) were present in 1 or more cyst preparations. A total of 60 various enzymes (including 19 protein kinases, 10 GTPases, 2 chitinase and a chitin synthetase) were also detected. Among other proteins, 8 vesicle coating or trafficking protein, 4 DNA repair proteins, 5 transcription or translation related proteins, 4 surface associated proteins, are notable.

The 417 *E. histolytica* proteins (Table S3) fell into several broad functional groups. About 28% (117 proteins) did not have a known function and are referred to as ‘hypothetical proteins’ (Figure 4). About 12.9% (54 proteins) contained putative transmembrane (TM) domains (Table S3). Perhaps the most important were the categories of 195 unique proteins that showed no overlaps with the trophozoite specific proteome or EST datasets (Figure 5). About 39.5% of these proteins (77 proteins) did not have a known function.

The *E. invadens* cyst wall is composed of chitin (a homopolymer of ß-1,4-linked GlcNAc), and chitin-binding Jacob and Jessie lectins and chitinases [37]–[39]. The prior transcriptome data from encysting cultures *in vitro* supported that these proteins were components of the *E. histolytica* cyst [22]. In this study, among the 195 unique cyst proteins, we identified one member of the Jacob gene family (EHI_028930), two chitinases (EHI_109890 and EHI_152170), and one chitin synthetase 2 (EHI_044840) supporting prior suppositions as to the composition of the cyst wall [22], [39]. Besides cyst wall proteins, 4 putative surface associated proteins were identified in the 195 cyst proteins. Three of these belonged to the BspA-like leucine rich repeat (LRR) protein family. An important aspect of BspA family proteins is to mediate protein-protein interactions [40]. In *E. histolytica*, more than 70 proteins belong to BspA-like LRR protein family, although only 4 of these were previously identified as being cyst-specific based on mRNA transcriptome data [22]. One BspA family member (EAL42510/849.m00008) was previously reported to be primarily located on the plasma membrane of *E. histolytica* trophozoite [41]. Consistent with the trophozoite-specificity of this protein, we could not identify this protein in the present cyst proteome. Another BspA family protein has been implicated in trifluoromethionine resistance [42]. Immunofluorescence studies with the episomal overexpression of this protein suggest that it is expressed in the cytoplasm of the *E. histolytica* trophozoites. Again, this protein was not identified in our cyst proteome, consistent with trophozoite-specificity. The other potential surface protein identified was a putative member of lectin family protein (EHI_110830).

Actin is one of the most conserved and ubiquitous proteins in eukaryotes. During encystation, morphological changes occur in the trophozoite structure of *E. histolytica*, and elongated trophozoites become spherical cysts. We anticipate a reorganization of the actin cytoskeleton during this process. One study shows that jasplakinolide, an actin-polymerizing and filament-stabilizing drug, inhibits both growth and encystation of *E. histolytica* and *E. invadens* through perturbation of the actin cytoskeleton [43]. Four actin or actin-related proteins were identified in the cyst proteome that might be involved in these morphological and structural changes, and deserve further studies.

Transmembrane domain containing protein kinases (TMKs) are involved in signal transduction in higher eukaryotes. Over 100 TMKs have been identified in the *E. histolytica* genome [44]. Since these proteins have extracellular domains coupled to cytoplasmic kinase domains, they have the potential to sense environmental cues. Fourteen of these TMKs showed significantly differential mRNA expression in encystating *E. histolytica* cultures compared to that of trophozoites [22]. Additionally, a number of cytoplasmic protein kinases have also been identified to be cyst-specific [22]. The *E. histolytica* genome encodes for about 343 protein kinases (including >100 TMKs), and 19 of these were identified in the 195 unique cyst proteins. Protein kinases regulate cellular pathways, especially those involved in signal transduction. More than half of these protein kinases (11/19) were predicted to have transmembrane domains, suggesting their potential roles in sensing outside clues for stage conversion (Table S3) [44].

GTPases are a large family of GTP-binding hydrolase enzymes involved in various biological functions including signal transduction, protein biosynthesis, cell division, translocation of proteins through membranes and transport of vesicles within the cell. The GTP-binding proteins are classified into five families – Ras, Rho, Rab, Arf and Ran [45]. Recent data suggest that the *E. histolytica* genome codes for 17, 22, 73, 12 and 1 member(s) of Ras, Rho, Rab, Arf and Ran proteins, respectively [45]. Additionally there are 41 unclassified small G proteins in the *E. histolytica* genome. The Ras proteins are mainly involved in cell proliferation. The Rho proteins are implicated in cytoskeleton regulation. Both Rab and Arf proteins are involved in membrane trafficking in the cytosol. The Ran proteins are implicated in nuclear-cytosol transport. It is anticipated that a dramatic change of cellular components occurs in a rapid, yet highly regulated fashion during encystation, involving *de novo* synthesis of new proteins and degradation of unwanted proteins. In order to achieve this, the amoeba is expected to have a developmental–specific system of membrane trafficking. The transcriptomic changes of Rab genes have been studied during the encystation of *E. invadens*, and 23 cyst-specific, 36-trophozoite-specific and 31 constitutively expressed Rab genes were identified [46]. In the 195 unique cyst proteins, we identified 10 GTPases (including members of Rab or related proteins) that may have stage-specific functions in *E. histolytica* (Figure 5; Table S3).

Four DNA repair proteins, including two DNA double-strand break repair proteins (EHI_053200 and EHI_125910), one DNA mismatch repair protein (EHI_126120) and a type A flavoproteins (EHI_129890) were identified in the 195 cyst proteins. We also found 8 vesicle coating (or trafficking) related proteins including 3 putative clathrin proteins which are generally involved in shaping rounded vesicles in the cytoplasm for intracellular trafficking [47]. In addition, a SET domain containing histone lysine methyltransferase (EHI_0319060) that adds methylmarks on histone tails, and two chromodomain-containing proteins (EHI_000780 and EHI_031370) that bind methylmarks on histone tails and facilitate the recruitment of transcriptional effector molecules were also identified, suggesting a role for epigenetic machineries in the development of *E. histolytica*.

### *E. histolytica* cyst proteins with diagnostic potential

Among various species of *Entamoeba* that can infect humans, only *E. histolytica* can cause intestinal and extraintestinal diseases in humans. At least two other human-infecting *Entamoeba* species, *E. dispar* and *E. moshkovskii*, are morphologically identical to *E. histolytica*. These commensal species create diagnostic challenges. Although *E. dispar* has never been documented to cause diseases, some emerging data suggest *E. moshkovskii* may cause diseases in humans [48], [49]. It is recommended that individuals with *E. histolytica* infection regardless of clinical status be treated due to the risk of the development of invasive amebiasis even in asymptomatic individuals. However, no simple *E. histolytica* cyst-specific diagnostic test is currently available to detect carriers. Here, we developed an immunofluorescence assay (IFA) using rabbit anti-Jacob antiserum that could detect *E. histolytica* cysts (Figure 1); however this polyclonal antibody might not be specific to *E. histolytica*. We conclude that proteins identified in this study are likely to be at least partially specific to *E. histolytica*, and therefore warrant further investigation as to their diagnostic potential.

In order to identify candidate diagnostic targets, we focused initially on the list of 195 unique proteins that were not detected in any trophozoite specific proteome or EST datasets. Twenty-five of these proteins showed 80% or less identity on the amino acid level compared to homologues in *E. dispar* (Table 1). These proteins displayed even lower identities with other proteins in the database including *E. moshkovskii* and human proteins. Over half (13/25) of the 25 proteins have no known functions and are annotated as hypothetical proteins. Five of these had previously shown 141- to 1012-fold higher mRNA transcript levels in cyst-like cultures compared to cultured trophozoite of *E. histolytica* [22]. Three out of 25 proteins in the list have no homologues in *E. dispar* including two that are putative reverse transcriptases (416.m00035 and 453.m00043) and one hypothetical protein (112.m00115).

**Table 1:** *E. histolytica* cyst proteins that show 80% or less identity with *E. dispar* proteins.

*E. histolytica* Locus ID	*Detected in samples	Annotation	Transcriptome data**			*E. dispar* homolog	
			p-value	fold change: cyst/troph	HM-1 expr	Locus ID	% identity
416.m00035	2	reverse transcriptase, putative	NF	NF	NF	None	N/A
453.m00043	2	reverse transcriptase, putative	NF	NF	NF	None	N/A
112.m00115	2, 4	hypothetical protein	NF	NF	NF	None	N/A
EHI_117680	3	protein kinase, putative	0.1170	2.9	0.05	EDI_160040	34
EHI_087690	3	hypothetical protein	0.0282	140.8	0.05	EDI_102140	44
EHI_075310	1, 2	hypothetical protein	0.2720	382.5	0.04	EDI_204760	47
EHI_182030	2	EhRabF2	0.1280	−1.7	1.96	EDI_154730	48
EHI_133790	1, 2	hypothetical protein	0.0161	84.9	0.04	EDI_063010	49
EHI_066720	4	MAEBL, putative	0.3900	−1.6	2.27	EDI_281920	54
EHI_019630	1, 4, 5	hypothetical protein	0.0001	515.8	0.08	EDI_199570	54
EHI_174540	5	Tyrosyl-DNA phosphodiesterase, putative	0.5250	1.6	0.04	EDI_259130	59
EHI_113200	1	hypothetical protein	0.3480	2.5	0.04	EDI_078590	59
EHI_047820	2	BspA-like leucine rich repeat protein, putative	0.2670	1.8	0.05	EDI_238280	63
EHI_104230	1	hypothetical protein	0.0001	1012.0	0.05	EDI_083080	64
EHI_146120	1, 2, 4	hypothetical protein	0.0001	59.1	0.04	EDI_105270	65
EHI_133780	1	hypothetical protein	0.0001	1012.0	0.05	EDI_083080	66
EHI_158620	1	Opioid growth factor receptor (OGFr) conserved region	0.3260	1.7	0.26	EDI_036800	69
EHI_059040	3	protein kinase domain containing protein	NF	NF	NF	EDI_142140	70
EHI_126120	1, 2	DNA mismatch repair protein mutL	0.4520	1.7	0.06	EDI_012590	72
EHI_034210	2	Protein kinase, putative	0.0450	−1.6	0.05	EDI_142140	72
EHI_174190	1, 2	hypothetical protein	0.1110	−3.4	0.54	EDI_018190	72
EHI_175920	4	protein kinase, putative	NF	NF	NF	EDI_280160	74
EHI_092210	3	hypothetical protein	0.1160	2.9	0.04	EDI_154030	75
EHI_163520	4	hypothetical protein 256.t00008	0.0563	−2.5	1.60	EDI_222270	80
EHI_138010	1	hypothetical protein	0.3790	1.9	0.20	EDI_134120	80

*: Detected in samples: 1 = 8076; 2 = 4268; 3 = AM951; 4 = AM797; and 5 = CMS33-7132;

**: From [22], 2007; NF = not found; troph = trophozoite; expr = expression; N/A = Not applicable. NB. During the revision of this manuscript, two new trophozoite-specific proteome datasets became available at the AmoebaDB on January 25, 2012. However, none of the proteins listed in Table 1 could be detected in any of the 4 trophozoite-specific proteome datasets available at the AmoebaDB or EST datasets (as of February, 2012).

For diagnostic purposes, proteins that were identified in a majority of the cyst samples are hypothesized to be consistently expressed in excreted cysts at detectable levels, and therefore potentially interesting as candidate diagnostic targets. Eight proteins were identified (from the list of 195 unique proteins) in 3 or more (out of 5) cyst samples (Table 2). Four of the 8 proteins displayed 90% or less amino acid level homologies with proteins from *E. dispar*. Two of these were putative cyst-wall specific proteins: chitinase (EHI_109890, which showed 87-fold higher mRNA transcript in encysting culture) and chitinase Jessie 3 (EHI_152170, which showed 44-fold higher mRNA transcript in encysting culture).

**Table 2:** *E. histolytica* cyst proteins detected in 3 or more cyst samples.

*E. histolytica* Locus ID	*Detected in samples	Annotation	Transcriptome data**			*E. dispar* homolog	
			p-value	fold change: cyst/troph	HM-1 expr	Locus ID	% identity
EHI_012500	3, 4, 5	coatomer gamma subunit, putative	0.0881	−2.7	1.90	EDI_129270	92
EHI_038630	2, 3, 4, 5	TBC domain containing protein, putative	0.3190	1.7	0.06	EDI_259490	91
**EHI_019630**	**1, 4, 5**	**hypothetical protein**	**0.0001**	**515.8**	**0.08**	**EDI_199570**	**54**
200.m00090	1, 4, 5	clathrin heavy chain, putative	0.7530	−1.0	32.75	EDI_237980	92
**EHI_109890**	**1, 3, 4, 5**	**chitinase, putative**	**0.0001**	**87.4**	**0.20**	**EDI_120190**	**82**
**EHI_152170**	**1, 3, 4, 5**	**chitinase Jessie 3, putative**	**0.0001**	**43.5**	**0.67**	**EDI_038370**	**90**
**EHI_146120**	**1, 2, 4**	**hypothetical protein**	**0.0001**	**59.1**	**0.04**	**EDI_105270**	**65**
EHI_196570	1, 2, 3, 4, 5	hypothetical protein	0.0001	37.2	0.55	EDI_039770	93

*: Detected in samples: 1 = 8076; 2 = 4268; 3 = AM951; 4 = AM797; and 5 = CMS33-7132;

**: From [22]; NF = not found; troph = trophozoite; expr = expression; N/A = Not applicable; shown in bold fonts are those that show 90% or less identity with *E. dispar* proteins; ideal candidates for *E. histolytica* specific diagnostic tool development. NB. None of the proteins listed in Table 2 could be detected in any of the 4 trophozoite-specific proteome datasets available at the AmoebaDB or EST datasets (as of February, 2012).

Proteins detected in our study have variable number of spectrum counts as detected by the LC-MS/MS experiments. For diagnostic purposes, proteins with greater numbers of spectrum counts may be better candidates for diagnostic assay development, as they may be more stable and more abundant in samples. Seven proteins were identified (from the list of 195 unique proteins) with more than 10 spectrum counts (Table 3). Four of the 7 proteins displayed 90% or less amino acid level identities with proteins from *E. dispar*. Two of these are chitinase (EHI_109890) and chitinase Jessie 3 (EHI_152170) with 52 and 24 spectrums detected, respectively, while the remaining two are hypothetical proteins (EHI_146120, 36 spectrums; and EHI_019630, 15 spectrums). Overall, Tables 1, 2, and 3 describe a total of 32 unique (non-redundant) proteins with diagnostic potential.

**Table 3:** *E. histolytica* cyst proteins with more than 10 spectrum counts detected in MS/MS experiments.

*E. histolytica* Locus ID	*Detected in samples	Annotation	Transcriptome data**			*E. dispar* homolog		No. of spectrum counts in LC-MS/MS
			p-value	fold change: cyst/troph	HM-1 expr	Locus ID	% identity	Total
10.m00319	4, 5	actin	0.2720	1.1	127.75	EDI_251490	100	193
**EHI_109890**	**1, 3, 4, 5**	**chitinase, putative**	**0.0001**	**87.4**	**0.20**	**EDI_120190**	**82**	**52**
**EHI_146120**	**1, 2, 4**	**hypothetical protein**	**0.0001**	**59.1**	**0.04**	**EDI_105270**	**65**	**36**
EHI_196570	1, 2, 3, 4, 5	hypothetical protein	0.0001	37.2	0.55	EDI_039770	93	36
**EHI_152170**	**1, 3, 4, 5**	**chitinase Jessie 3, putative**	**0.0001**	**43.5**	**0.67**	**EDI_038370**	**90**	**24**
**EHI_019630**	**1, 4, 5**	**hypothetical protein**	**0.0001**	**515.8**	**0.08**	**EDI_199570**	**54**	**15**
200.m00090	1, 4, 5	clathrin heavy chain, putative	0.7530	−1.0	32.75	EDI_237980	92	11

*: Detected in samples: 1 = 8076; 2 = 4268; 3 = AM951; 4 = AM797; and 5 = CMS33-7132;

**: From [22]; NF = not found; troph = trophozoite; expr = expression; N/A = Not applicable; shown in bold fonts are those that show 90% or less identity with *E. dispar* proteins; ideal candidates for *E. histolytica* specific diagnostic tool development.

NB. Only a single spectrum was detected for a high proportion of 195 cyst proteins (137 or 70%) compared to that detected in 914/1825 (or 50%) of trophozoite MS/MS proteins deposited into the AmoebaDB database (http://amoebadb.org/amoeba/). In fact, other proteins other than *E. histolytica* in the cyst samples hampered an efficient identification of cyst specific proteins. The sources of these other proteins include (1) numerous bacteria that were attached to the surface of the cyst, or ingested by the cyst, and (2) the human feces, which was the original source of cyst material contributed the human proteins and diet-related (mainly rice) proteins. In contrast, the trophozoite MS/MS data in the AmoebaDB was coming from two studies that used axenically grown *E. histolytica* trophozoites, which should be free from all other organisms including bacteria [29], [30]. Also, note that none of the proteins listed in Table 3 could be detected in any of the 4 trophozoite-specific proteome datasets available at the AmoebaDB or EST datasets (as of February, 2012).

In addition to candidate proteins for cyst specific diagnosis, we also identified proteins in the remaining 222 proteins out of the 417 (that were also present in the trophozoite specific proteome and EST datasets). These may also be useful to develop diagnostic tools to detect both cysts and trophozoites of *E. histolytica*. These proteins are listed in Tables S4, S5, S6.

### Host (human) proteins and bacterial species identified in at least 3 out of 5 cyst samples

Thirty-one human proteins were identified in at least 3 out of 5 cyst samples including 7 that were identified across all 5 samples, and 10 that were identified in 4 samples (Table S7). Multiple lectin- or sugar-binding proteins such as galectin-3 (IPI00023673), galectin-4 (IPI00009750), intelectin-1 (IPI00291737), intelectin-2 (IPI00103436), glycoprotein 2 (IPI01014468), and proteoglycan 3 (IPI00005778) were detected. The possible interaction of some of these proteins with the cyst or trophozoite surface and their potential roles in stage-conversion may warrant further investigation. Additional proteins detected in the cyst samples derived from bacterial origins (Table S8).

### Annotation of the genome of *E. histolytica*

The *E. histolytica* gene annotation is limited at present, although it is improving through the combined efforts of biostatisticians and researchers [50]. More than half of the genes (53.8%; 4413 genes out of a total predicted 8201 genes) are designated as “hypothetical” proteins of unknown function [50]. This is mainly because the *E. histolytica* genes are highly diverse in sequence compared to other organisms, which makes prediction of gene function difficult. One hundred and seventeen (or 28%) of 417 proteins identified in this study were predicted “hypothetical” proteins including 40 that were previously identified in trophozoite derived proteome or EST datasets. For the remaining 77 cyst specific “hypothetical” genes we now have evidence of protein level expression. We also found that 9 out of 417 protein genes identified in this study were missing in the most recent genome annotation (http://amoebadb.org/amoeba/). Three of these are putative clathrin heavy chain containing proteins (gi|103484580, found in sample AM951; 200.m00090, found in samples 8076, AM797, CMS33-7132; and 141.m00078, found in sample AM797) that are involved in vesicle trafficking in other organisms. Additional non-annotated genes encode 2 putative reverse transcriptases (416.m00035 and 453.m00043, both found in sample 4268), a putative Sec61 alpha subunit (gi|52352493, found in sample AM951), an actin (10.m00319, found in samples AM797 and CMS33-7132), and two hypothetical proteins (112.m00115, found in samples 4268 and AM797; and 270.m00054, found in sample CMS33-7132). We now have evidence that these genes are expressed as proteins in *E. histolytica* cysts.

## Discussion

Previous studies characterized gene expression in *E. histolytica* and *E. invadens* cells that have encysted under laboratory conditions. The goal of the present study was to obtain the proteomic profiles of *E. histolytica* cells that have encysted under natural conditions. To achieve this we employed a highly sensitive, whole genome shotgun sequencing approach using mass spectrometric methods. We successfully identified 417 proteins representing 5.1% of all predicted proteins in *E. histolytica*. Of these 417 proteins 191 overlapped with the proteins previously identified in trophozoite proteome. Similarly, 109 proteins overlapped with the previously identified trophozoite specific EST datasets (Figure 3). The remaining195 proteins have not been seen in trophozoite proteomes and are likely to be specific to cyst stage of the parasite.

Analysis of each of the cyst preparations identified 195 unique proteins, many of which warrant further investigation as potential diagnostic targets. The 417 identified proteins comprise approximately 5.1% of all predicted proteins in *E. histolytica*, suggesting that the proteomic data constitutes a partial representation of all proteins present in cysts. However, we cannot know the true percentage of representation since the total number of proteins upregulated during encystation is not known. Part of the difficulty in identifying cyst-specific proteins lies in part to the low proportion of *E. histolytica* proteins in the Percoll-purified cyst samples, with ≥90% of the proteins identified of human, rice, bacteria or other origin. An improved method of separation of *E. histolytica* cysts from other materials present in stool samples should be considered in future proteomic work. Although this study may not represent a comprehensive proteome of *E. histolytica* cysts, it succeeded in identifying some of the most abundant proteins in the naturally occurring cysts. These proteins will likely be useful targets for developing improved diagnostic tests for *E. histolytica* infection, as demonstrated by the ability to identify cysts in fecal specimens using antibodies directed to the cyst-specific Jacob protein.

There were several sample-based and technical limitations in this work. First, 5 asymptomatically infected 2–6 year-old children provided all the *E. histolytica* cyst positive stool samples. As a result, the amount of stool was small in each case. Efforts to collect additional samples from these patients were not successful as the number of cysts usually decreased in successive stools. Second, due to the nature of source material (human stool), unwanted host proteins, diet related (e.g., rice) proteins, and bacterial proteins co-purified with cysts despite our efforts to reduce these by using vigorous washing steps. Bacterial proteins may have been derived from bacteria attached to the surface of cysts; these would be extremely difficult (if not impossible) to exclude. Overall, the presence of contaminating proteins compromised the identification of *E. histolytica* cyst proteins. An alternate approach to Percoll gradient purification (such as CsCl gradient) should be considered in future cyst purification. However, despite these technical limitations, 195 novel *E. histolytica* proteins were still identified marking a significant advancement in our knowledge of the cyst proteome.

The *E. histolytica* cyst is a biodefense threat to water and food supplies due to its resistance to chlorination and low infectious dose (<10 cysts). It is also a public health threat, especially as a cause of diarrhea in children in Africa, Asia, and Latin America. However, the presence of morphologically indistinguishable cysts of non-pathogenic species *E. dispar* and *E. moshkovskii* severely complicates current microscopy based diagnosis which is neither sensitive nor specific. *E. histolytica* infections lead to intestinal and extra-intestinal amebiasis. Each year 40 to 50 million cases of colitis and liver abscess due to the pathogenic *E. histolytica* occur, causing up to 100,000 deaths. So, the development of sensitive molecular based techniques to detect *E. histolytica* cyst is a priority. In this study, we have been successful in identifying several target proteins that may lead to develop improved diagnostic tools to detect cysts in asymptomatic cyst passers (Tables 1, 2, 3). It is worth mentioning here that two new trophozoite-derived proteome works became available at the AmoebaDB public database (on January 25, 2012) while this manuscript was under revision [51], [52]. However, none of the 32 (non-redundant) cyst-specific candidate proteins listed in Tables 1, 2, and 3 could be detected in the new trophozoite-derived proteomic datasets. So, we are confident that some of these proteins would be highly useful as cyst-specific diagnostic targets. In addition, some of the proteins identified in this study will help to develop diagnostic tools capable of detecting both cyst and trophozoite forms of the parasite (Tables S4, S5, S6). A recent study shows that sera of amebic liver abscess patients can recognize a 110 kDa protein (annotated as pyruvate phosphate dikinase, EHI_009530) [53]. This protein was identified in all 5 cyst samples in our study (see Table 2 or Table S3), supporting the notion that proteins identified in this study have promise as candidate diagnostic targets.

Our knowledge about the cyst stage of *E. histolytica* is very limited due in part to our inability to induce encystation *in vitro*. We do not know if the involvement of host proteins is a necessary factor. It appears likely that bacterial involvement is essential for encystation to take place as encystation cannot be induced in axenic (bacteria-free) culture. However, we do not know the mechanism of bacterial association in encystation. For example, we do not know if certain bacterial species are essential or if certain bacterial proteins can efficiently induce encystation. Carefully designed functional studies using some of the human and bacterial proteins detected in this study (Tables S7, S8) may provide clues as to their involvement in encystation of *E. histolytica*.

This study provides evidence of protein level expression of several genes that are missing in the current gene annotation. Likewise, this study also provides evidence of expression at the protein level for over a hundred genes that were annotated as “hypothetical” in the current gene annotation. Therefore these data will help improve the present gene annotation. These data will be made publicly available through the AmoebaDB.

The data generated may aid the understanding of biochemistry and physiology of cysts and the developmental switch between the trophozoite and cyst stage in *E. histolytica*, a process that is vital for disease transmission and pathogenesis in this parasite. Among the cyst specific 195 proteins, it is intriguing to find numerous transmembrane domain containing protein kinases, vesicle coating or trafficking related proteins, DNA repair proteins, and GTPase-based signal molecules in the present study. Further functional work on these cyst proteins may provide insight into the signaling pathways that trigger encystation, as well as mechanisms of stage conversion, which will aid in developing better therapeutic agents and preventive measures to control amebiasis.

### List of genes/proteins discussed in this paper

***E. histolytica*** **:** EHI_044500, EHI_065330, EHI_109890, EHI_152170, EHI_197480, EHI_130700, EHI_028930, EHI_044840, EAL42510, EHI_110830, EHI_199590, EHI_053200, EHI_125910, EHI_126120, EHI_129890, 416.m00035, 453.m00043, 112.m00115, EHI_146120, EHI_019630, gi|103484580, 200.m00090, 141.m00078, gi|52352493, 10.m00319, 270.m00054, EHI_0319060, EHI_000780 and EHI_031370. ***E. invadens*** **:** EiChit1, EiChit4, AF252299/BiP, EIN_093390, EiCP-B9, EiPFN1, and EiPFN4. **Human:** galectin-3/IPI00023673, galectine-4/IPI00009750, intelectin-1/IPI00291737, intelectin-2/IPI00103436, glycoprotein 2/IPI01014468, and proteoglycan 3/IPI00005778.

## Supporting Information

Figure S1 **Total number of** ***E. histolytica*** **proteins detected in soluble, insoluble or both fractions of mass spectrometry experiments for 4 cyst samples.** The mass spectrometry was carried out for 4 out of 5 cyst samples in such a way that it could detect both soluble and insoluble proteins (for details, see the Methods section). For the 5^th^ sample (4268), there was not enough cyst material to proceed by this method, and proteins identified in this sample represented both soluble and insoluble proteins. Except for the sample 8076 (which has a protein distribution such as 48 in soluble fraction, 43 in insoluble fraction, and 18 in both fractions), there was a general trend that relatively more proteins were identified in the insoluble fraction compared with the soluble fraction (59/48 for AM951, 99/42 for AM797, and 57/35 for CMS33-7132).
(TIF)

Figure S2 **Overlap between the 417 proteins (from this study) and the cyst-specific mRNA transcripts (from** [**22**] **).** The overlap between 672 cyst-specific transcripts (p-value<0.05 and fold-change ≥3) and 394 proteins out of all 417 proteins from cyst proteomic study (except for the remaining 23 proteins, that were not found in microarray data) was tested using the Venn diagram. The overlap between the cyst protein data and the cyst-specific mRNA transcript data was statistically significant (p-value 0.0058). The p-value was determined using the two-tailed Fisher's exact test using the GraphPad software freely available in the internet at http://www.graphpad.com/quickcalcs/contingency1.cfm.
(TIF)

Figure S3 **Overlap between the 195 cyst –specific proteins (from this study) and the cyst-specific mRNA transcripts (from** [**22**] **).** The overlap between 672 cyst-specific transcripts (p-value<0.05 and fold-change ≥3) and the 185 proteins out of 195 proteins from the cyst proteomic study that were not identified in trophozoite-specific proteome or EST datasets (except for the remaining 10 proteins, that were not found in the microarray data) was tested using the Venn diagram. The overlap was statistically significant (p-value 0.0014). The p-value for the overlap of this comparison is better than the previous comparison shown in Figure S2 (0.0014 versus 0.0058, respectively) as expected. The p-values were determined using the two-tailed Fisher's exact test using the GraphPad software freely available in the internet at http://www.graphpad.com/quickcalcs/contingency1.cfm.
(TIF)

Table S1 **Proteins detected in sample AM951 using two different processing methods prior to LC-MS/MS experiments.**
(XLSX)

Table S2 **Categories of proteins identified in sonication only and freeze-thaw-sonication, or both approaches for sample AM951.**
(XLSX)

Table S3 **All the** ***E. histolytica*** **proteins detected in 5** ***E. histolytica*** **cyst samples (N = 417).**
(XLSX)

Table S4 ***E. histolytica*** **proteins detected in cyst samples that show 80% or less identity with** ***E. dispar*** **proteins.** These are candidate proteins for development of diagnostic tool for both trophozoites and cysts of *E. histolytica*.
(XLSX)

Table S5 ***E. histolytica*** **proteins detected in 3 or more of 5 cyst samples.** These are candidate proteins for development of diagnostic tool for both trophozoites and cysts of *E. histolytica*.
(XLSX)

Table S6 ***E. histolytica*** **proteins with maximum number of spectrum counts detected in cyst samples by LC-MS/MS experiments.** These are candidate proteins for development of diagnostic tool for both trophozoites and cysts of *E. histolytica*.
(XLSX)

Table S7 **Host (human) proteins detected in cyst samples.** Human proteins that were identified in at least 3 out of 5 *E. histolytica* cyst samples are shown in this Table.
(XLSX)

Table S8 **Bacterial species detected in 3 or more** ***E. histolytica*** **cyst samples.** Many bacterial peptides were identified in the *E. histolytica* cyst samples by LC-MS/MS experiments. From the database search using the peptide sequences, the bacterial species were identified. At least 19 bacterial species were detected in 3 or more *E. histolytica* cyst samples.
(XLSX)
