# Supplementary figures and images for: Proteomic Analysis of the Cyst Stage of Entamoeba histolytica
Source: PLoS Negl Trop Dis. 2012 May 8;6(5):e1643. doi: 10.1371/journal.pntd.0001643 (PMC3348168; doi:10.1371/journal.pntd.0001643)

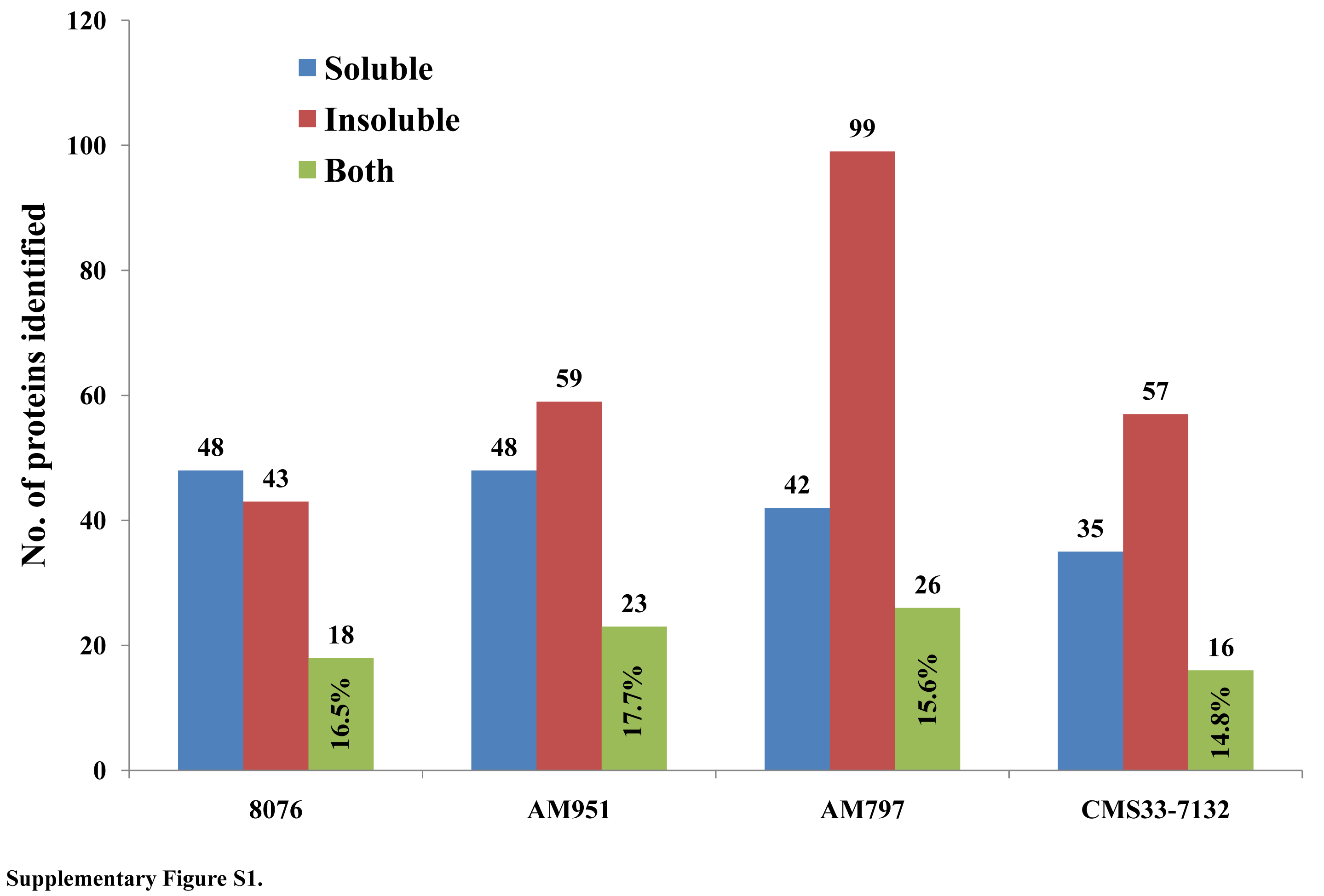

Supplement: Figure S1 — Total number of E. histolytica proteins detected in soluble, insoluble or both fractions of mass spectrometry experiments for 4 cyst samples. The mass spectrometry was carried out for 4 out of 5 cyst samples in such a way that it could detect both soluble and insoluble proteins (for details, see the Methods section). For the 5th sample (4268), there was not enough cyst material to proceed by this method, and proteins identified in this sample represented both soluble and insoluble proteins. Except for the sample 8076 (which has a protein distribution such as 48 in soluble fraction, 43 in insoluble fraction, and 18 in both fractions), there was a general trend that relatively more proteins were identified in the insoluble fraction compared with the soluble fraction (59/48 for AM951, 99/42 for AM797, and 57/35 for CMS33-7132). (TIF) [file pntd.0001643.s001.tif]

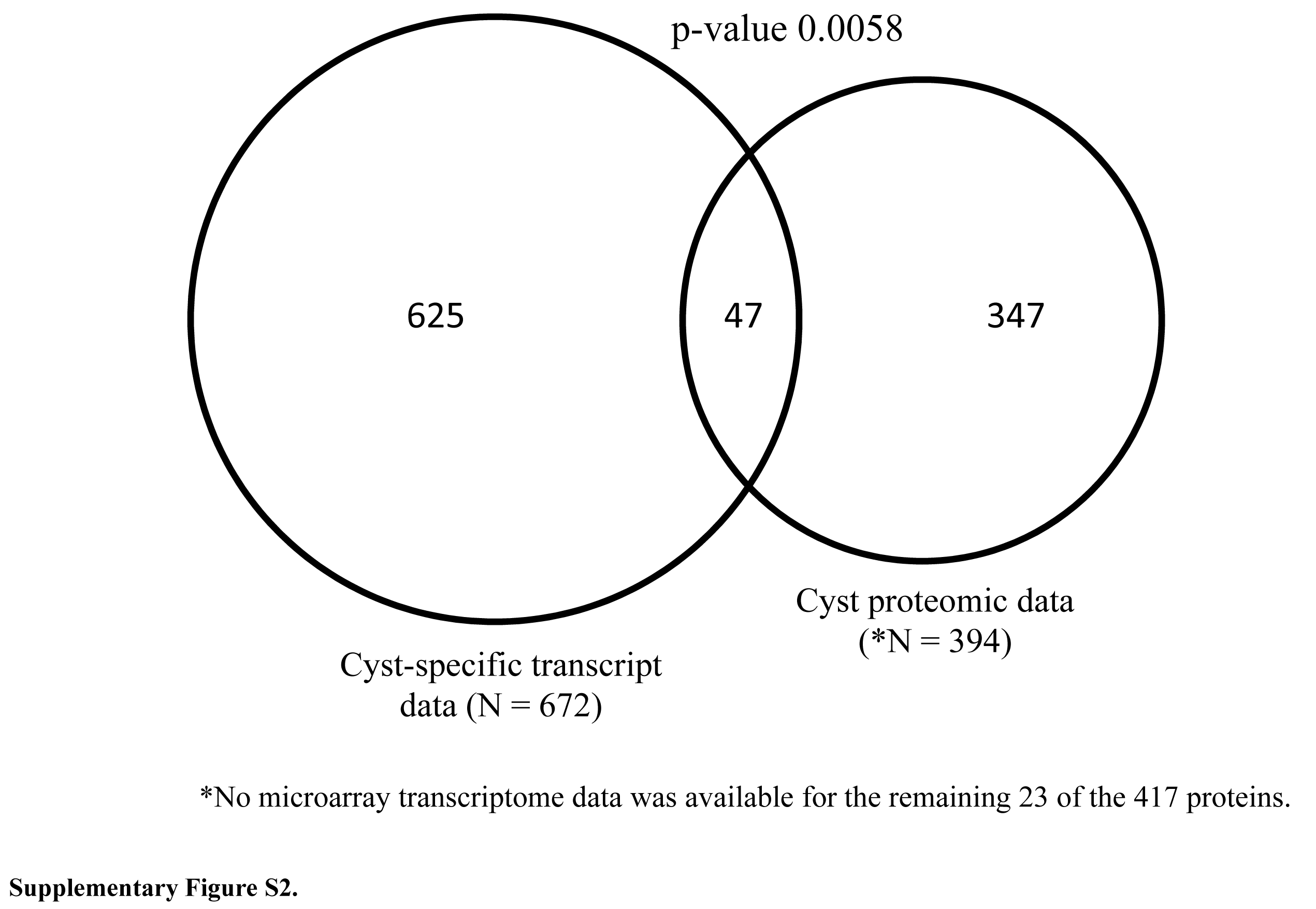

Supplement: Figure S2 — Overlap between the 417 proteins (from this study) and the cyst-specific mRNA transcripts (from [22] ). The overlap between 672 cyst-specific transcripts (p-value<0.05 and fold-change ≥3) and 394 proteins out of all 417 proteins from cyst proteomic study (except for the remaining 23 proteins, that were not found in microarray data) was tested using the Venn diagram. The overlap between the cyst protein data and the cyst-specific mRNA transcript data was statistically significant (p-value 0.0058). The p-value was determined using the two-tailed Fisher's exact test using the GraphPad software freely available in the internet at http://www.graphpad.com/quickcalcs/contingency1.cfm. (TIF) [file pntd.0001643.s002.tif]

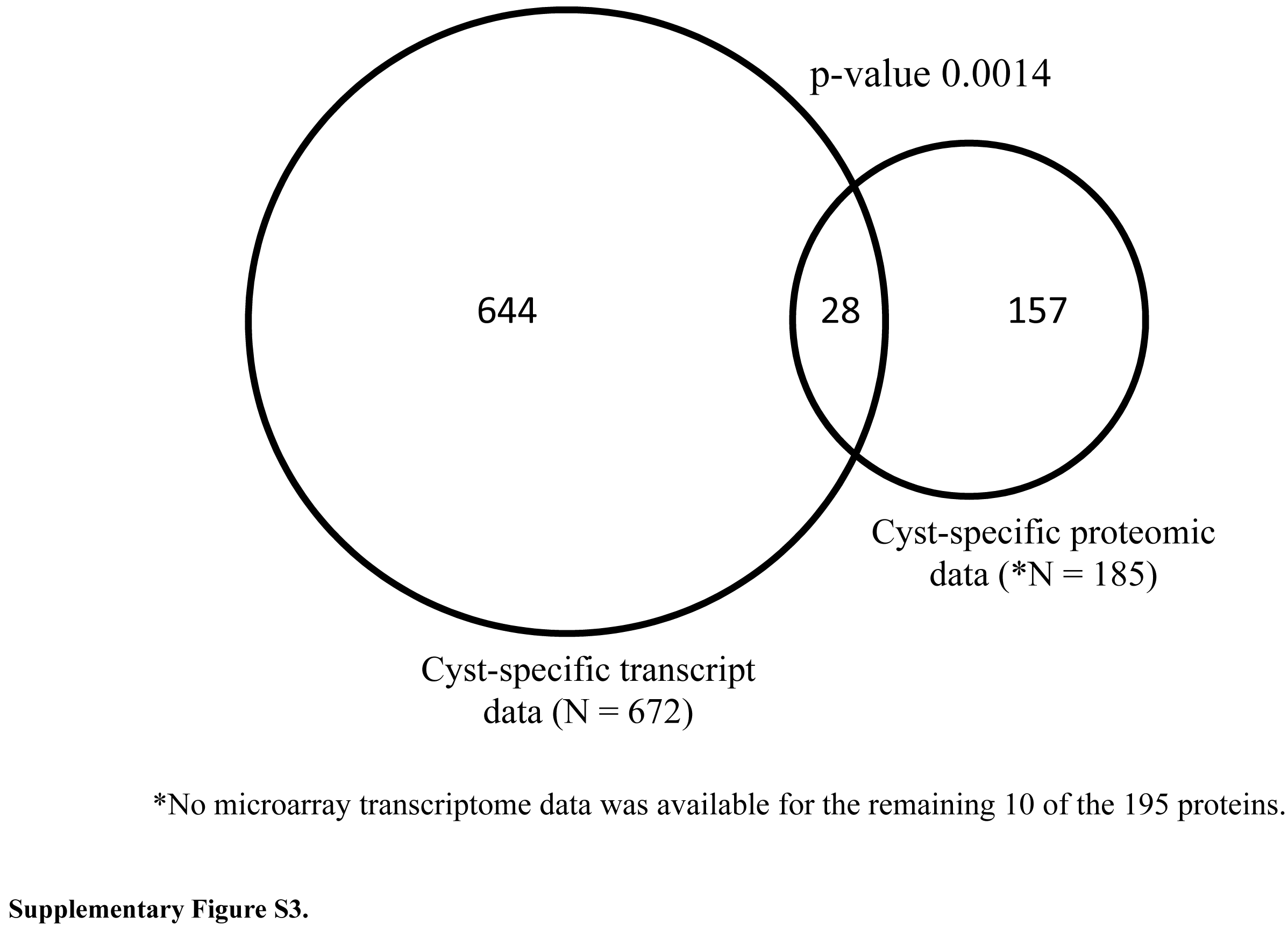

Supplement: Figure S3 — Overlap between the 195 cyst –specific proteins (from this study) and the cyst-specific mRNA transcripts (from [22] ). The overlap between 672 cyst-specific transcripts (p-value<0.05 and fold-change ≥3) and the 185 proteins out of 195 proteins from the cyst proteomic study that were not identified in trophozoite-specific proteome or EST datasets (except for the remaining 10 proteins, that were not found in the microarray data) was tested using the Venn diagram. The overlap was statistically significant (p-value 0.0014). The p-value for the overlap of this comparison is better than the previous comparison shown in Figure S2 (0.0014 versus 0.0058, respectively) as expected. The p-values were determined using the two-tailed Fisher's exact test using the GraphPad software freely available in the internet at http://www.graphpad.com/quickcalcs/contingency1.cfm. (TIF) [file pntd.0001643.s003.tif]
